# Letter Position Dyslexia in Fingerspelling: Similar Error Patterns in Reading Written and Fingerspelled Words

**DOI:** 10.3390/bs16050654

**Published:** 2026-04-26

**Authors:** Naama Friedmann, Neta Haluts, Doron Levy

**Affiliations:** Language and Brain Lab, School of Education and Sagol School of Neuroscience, Tel Aviv University, Tel Aviv-Yafo 6997801, Israel; nhaluts@gmail.com (N.H.); doronle1@gmail.com (D.L.)

**Keywords:** letter position dyslexia, sign language, fingerspelling

## Abstract

Letter position dyslexia is a deficit in letter position encoding in the orthographic-visual analysis stage. We report here the first cases of deaf signers who show letter position dyslexia in both written and fingerspelled words. Their error pattern was identical in the two modes of presentation, written and fingerspelled: in both modes, they had almost only within-word letter transpositions; their transpositions involved middle letters and almost never exterior letters; they had errors of doubled letters; and their errors occurred almost only in migratable words. They showed no transpositions in reading multi-digit numbers. These results indicate that despite the temporal separation between the fingerspelled letters, the reading of fingerspelling uses the same cognitive processes, and specifically, the same letter-position-encoding mechanism, as the reading of written words.

## 1. Introduction

**Figure 1:**
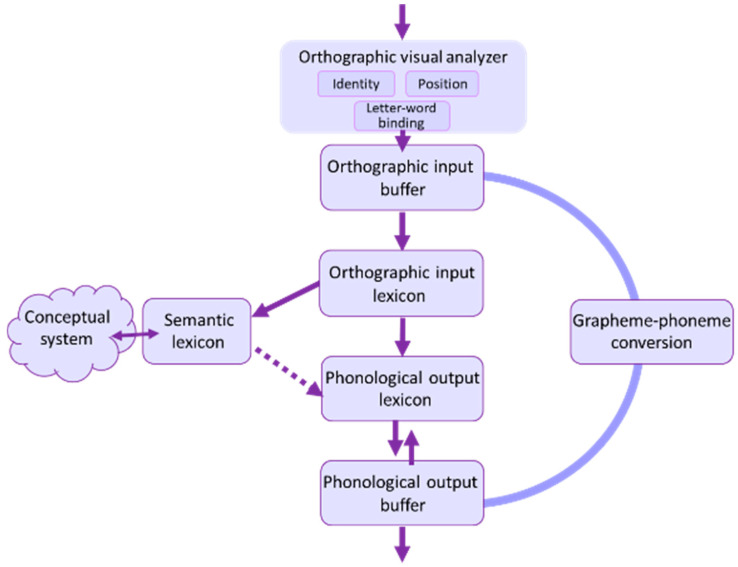
Stages of reading words and pseudo-words.

Reading is a multi-staged process (Castles, 2006; Coltheart, 2006a; Coltheart et al., 2001; Friedmann & Coltheart, 2018). This study focuses on the early stage of this process, the visual-orthographic analysis of the letter string (Figure 1). Specifically, we explore impairment in one process that takes place in the early stage: letter position encoding. We examine this component via the study of the way a deficit in letter position encoding is manifested in reading fingerspelled words and in reading written words.

### 1.1. The Reading Process and Types of Dyslexia

The initial reading stage, the orthographic-visual analyzer, involves three processes: identification of the individual letters, analysis of their relative positions within the word, and binding of the letters to the word in which they appear. This information is then held in a short-term orthographic buffer, which is also in charge of graphemic and morphological analysis. The following stage is the orthographic input lexicon, in which familiar words are retrieved from the long-term store of written word forms. From the orthographic input lexicon, information proceeds to semantic and conceptual systems, enabling the comprehension of the written word. The orthographic input lexicon is also connected to phonological output components—the phonological output lexicon and the phonological output buffer (POB)—which participate in the production of the word. Pseudo-words (and real words) can also proceed from the orthographic input buffer through the sub-lexical route, where they undergo grapheme-to-phoneme conversion, which ends in the POB (Friedmann & Coltheart, 2018).

In line with the different stages of reading, neuropsychological research from the last few decades revealed that dyslexia, an impairment in reading, is not a single deficit. Rather, there exist several different impairments in different stages of the reading process that lead to different types of specific reading impairments, which in turn lead to different patterns of errors (Beauvois & Dérouesné, 1979; Bub et al., 1985; Funnell, 1983; Marshall & Newcombe, 1973; McDougall et al., 2005). As many as 22 types of dyslexia have been identified (Coltheart & Kohnen, 2012; Friedmann & Coltheart, 2018; Friedmann & Haddad-Hanna, 2014; Güven & Friedmann, 2022; Potier Watkins et al., 2023; Traficante et al., 2024); each can be associated with specific stages in the dual-route model.

### 1.2. LPD and What We Know About It in Hearing Speakers

One specific type of dyslexia that has been identified is letter position dyslexia (LPD). LPD is an impairment in the visual-orthographic analyzer in the encoding of the letter position within the word, which has been demonstrated both in acquired and in developmental cases (Friedmann & Gvion, 2001, for the first report on an acquired case; Friedmann & Rahamim, 2007, for the first developmental case). Individuals with LPD transpose letters within words, especially the middle letters, mainly in migratable words (words in which transposition of the middle letters creates another existing word, e.g., files → flies and signer → singer). They also make omission and insertion errors with letters that appear twice in the word (non-geminates, e.g., drivers → divers; starts → stars). Friedmann and Rahamim (2007) suggested that such errors with doubled letters occur in LPD because doubled letters differ only in their position. When position encoding is impaired, the reader loses the only property that distinguishes between the two instances of the same letter, leading to errors such as omitting a doubled letter or doubling a single letter.

LPD has already been identified in English, Hebrew, Italian, Arabic, French, and Turkish (Friedmann & Gvion, 2001; Friedmann & Haddad-Hanna, 2012; Friedmann & Rahamim, 2007; Güven & Friedmann, 2019; Kohnen et al., 2012; Potier Watkins et al., 2023; Traficante et al., 2024), but until now, it had never been reported in deaf signers of any sign language, nor was it tested in the reading of fingerspelling.

### 1.3. Sign Language and Fingerspelling

Sign languages are natural languages, with unique grammars, separate from the grammars of the spoken languages in their environment (Cecchetto, 2016; Geraci, 2015; Meir & Sandler, 2007). Signs in sign languages are composed of smaller visual elements corresponding to spoken language phonology, such as handshape, movement, and place of articulation (or location in space) (Stokoe, 1960/2005). As sign languages do not have their own writing systems (Quer & Steinbach, 2019), deaf signers learn to read the orthographic system of the spoken language surrounding them (which in many cases is not their native language). Many sign languages also have systems of fingerspelling—a set of handshapes representing the orthography of the surrounding spoken language—which is used to spell out words that do not have a corresponding sign (e.g., names of people or places or borrowed common nouns—Padden & Gunsauls, 2003; Sutton-Spence & Woll, 1999).

**Figure 2:**
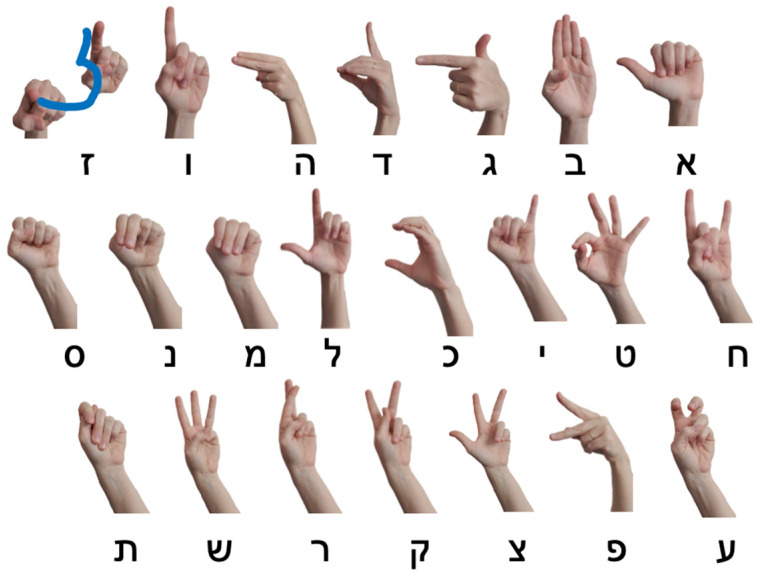
The fingerspelled alphabet in ISL. Printed letters show the parallel letter in the Hebrew orthography.

In Israeli Sign Language (ISL), the sign language spoken by the Deaf community in Israel, the fingerspelled alphabet corresponds to the Hebrew orthography. Interestingly, many of the fingerspelled letters in ISL do not resemble the Hebrew letters visually, since they are borrowed from the fingerspelling system of American Sign Language (ASL), with some adaptations (Meir & Sandler, 2007; see Figure 2).

Fingerspelled words are produced letter by letter, with temporal separation between the letters. Research and reading instruction practice consider fingerspelling important for deaf signers not only as a means to convey words that do not have a sign but also as an effective way to teach reading to deaf signers, as a bridge between the signed and spoken language (Blackburn et al., 1984; Crume, 2013; Haddad-Hanna, 2025; Haptonstall-Nykaza & Schick, 2007; Hirsh-Pasek, 1986; MacGlaughlin, 2018; Miller et al., 2021; Puente et al., 2006; Stone et al., 2015; Tucci et al., 2014). The process of fingerspelling and reading fingerspelled words (finger-reading) has never been studied in a neuropsychological approach; types of dyslexia in finger-reading have never been identified; and cognitive stages necessary for finger-reading have never been incorporated into neuropsychological models of reading.

Nevertheless, there are reasons to believe that similar mechanisms underlie the reading of written and fingerspelled words in deaf signers. The stage that is expected to differ the most between written and fingerspelled word reading is the orthographic-visual analysis stage, the early stage of the reading process. However, brain research has actually shown that finger-reading engages the Visual Word Form Area (VWFA), a region that is involved in the early orthographic-visual processing of written words (Bolger et al., 2005; Cohen et al., 2000, 2002). This indicates that similar brain regions process orthographic input, regardless of the modality of input (Waters et al., 2007). Furthermore, research has shown that there is a correlation between reading skills for written words and performance in finger-reading (Puente et al., 2006), that there exist orthographic plausibility effects similar to the ones known for written words when reporting the letters of finger-read words (Hanson, 1981), and that similar priming and short-term memory effects influence both written and fingerspelled words (Lee et al., 2025; Sehyr et al., 2016; Williams et al., 2015).

### 1.4. Testing LPD in Signers Can Help Decide Between Theories

The existence of the fingerspelling system in sign language enables researchers to learn both about the nature of specific types of dyslexia, and about the cognitive mechanisms underlying finger-reading. 

The way a type of dyslexia is manifested in finger-reading can reveal the properties of this dyslexia and the exact nature of the deficit leading to it. Specifically, finger-reading can contribute to a better understanding of the cognitive processes underlying the early stages of orthographic-visual analysis in reading and of dyslexias that result from a deficit in this stage. The fact that fingerspelled letters have temporal, rather than spatial, separation can help answer theoretical questions regarding the nature of letter position encoding and migration errors in reading. Friedmann and Gvion (2001) claimed that the deficit is orthographic, in the letter-encoding function. They also backed up this proposal by showing that individuals with LPD do not transpose numbers (see also Dotan & Friedmann, 2019, and Friedmann et al., 2010a, for dissociations between number and word reading). However, there are alternative proposals linking LPD to general visual failures (e.g., Stein, 2019). One explanation given for LPD is a deficit in visuo-spatial separation—or crowding (Gori & Facoetti, 2015; Martelli et al., 2009; Spinelli et al., 2002). Other proposals refer to lateral interference (see Friedmann & Gvion, 2001 for a discussion of this possibility). If we find that LPD is expressed similarly in fingerspelling, despite the fact that fingerspelled words are not presented visually together but are rather presented sequentially letter by letter, this would indicate LPD is an orthographic-specific failure rather than a general visual failure. Such findings would suggest that this deficit is tightly related to the processing of letter sequences and specifically to the encoding of letter positions within words.

Complementarily, studying dyslexia in finger-reading can be instrumental for understanding the cognitive mechanisms underlying finger-reading. This can be done via the exploration of the manifestation of the same dyslexia, in the same person with dyslexia, in two orthographic systems: the written and the fingerspelled systems, representing the same words. This can help us better understand the mechanisms underlying finger-reading and reveal whether the same cognitive mechanisms are responsible for the process of reading written and fingerspelled words, supporting their generality and independence from stimulus mode.

Two alternative parallels could be imagined with respect to the cognitive mechanisms of the reading of fingerspelling: one would be that reading a fingerspelled word is parallel to reading a written word. The other is that reading a fingerspelled word is parallel to reading written letters presented one by one. Friedmann and Rahamim (2014) showed that in LPD, reading each written letter separately reduces transposition rate in comparison to reading a whole word. This provides us with a tool to examine the question of parallelism and identify the written parallel of reading a fingerspelled word. Because the letters are signed one after the other rather than presented in parallel, if LPD in finger-reading is less pronounced, this would indicate that reading fingerspelled words is parallel to reading written letters presented one by one. Alternatively, if similar transposition rates are seen in the reading of fingerspelled and written words, this would indicate that finger-reading is parallel to reading whole written words and that the letter-position-encoding deficit in LPD affects an orthographic stage in which the word is represented as a whole.

In writing, we have recently shown that a Hebrew–ISL bilingual with orthographic (graphemic) output dysgraphia showed exactly the same pattern of errors with handwriting and fingerspelling (Haluts et al., 2025). We interpreted this result as indicating similar cognitive mechanisms for handwriting and fingerspelling. A parallel examination for reading, assessing whether the cognitive mechanisms are independent of modality in reading as well, has never been done in individuals with dyslexia.

### 1.5. Dyslexia in Deaf Individuals

A large body of research has been devoted to difficulties in reading and reading comprehension of deaf individuals (Allen, 1986; Beal-Alvarez et al., 2012; Harris et al., 2017; Holt, 1993; Lederberg et al., 2013; Luckner & Handley, 2008; Schirmer & McGough, 2005; Tomazin et al., 2025; Traxler, 2000). It seems that in general, reading at the word level for deaf individuals is similar to the experience of their peers (Burden & Campbell, 1994; Fischler, 1985; Wauters et al., 2006). Difficulties in reading comprehension, which are typically assessed at the text rather than word level, may be a result of language difficulties, such as reduced lexical knowledge of the spoken language and difficulties with syntax (Marschark et al., 2009; Padden & Ramsey, 1998; Szterman & Friedmann, 2020; Tomazin et al., 2025; Wauters et al., 2006), which have been shown to be present in deaf and hard-of-hearing children, independently of reading (Delage & Tuller, 2007; Friedmann & Haddad-Hanna, 2014; Friedmann & Szterman, 2006, 2011; Haddad-Hanna & Friedmann, 2009; Friedmann et al., 2010b; Volpato & Adani, 2009).

In contrast, there are very few reports of impairments specific to reading single words and pseudo-words in deaf children or adults, no neuropsychological case reports at all of specific types of dyslexia in deaf signers, and no reports of specific types of dyslexia in reading fingerspelled words.

One study that did report dyslexia in deaf signers was Enns and Lafond (2007). They reported two cases of deaf signers with dyslexia; however, they focused only on the number of correctly read words and did not specify the pattern of errors their participants exhibited, nor the specific type of dyslexia they had.

We have previously reported a deaf signer with a very specific impairment in reading-then-signing multi-digit numbers, who showed a dissociation between impaired reading of numbers and preserved reading of words (Friedmann et al., 2021), and another (hearing) aphasic signer who showed a selective and specific impairment in spelling, expressed both in handwriting and in fingerspelling, without an impairment in reading per se (Haluts et al., 2025); both cases show it is possible to dissociate different types of impairments in users of a sign language and support the need to identify specific impairments in the deaf signing population.

One possible explanation for the absence of reports of specific types of dyslexia in deaf signers is the lack of reading assessment tools suitable for deaf signers. For many deaf signers, speech production does not reflect their reading ability well enough. Using tasks of reading aloud may lead to errors related to speech production rather than to reading and lead to underestimation of their reading abilities. A reading-then-signing task, which we use in our reading assessments of deaf signers and in the current study, avoids this issue, and also ensures that the origin of the transposition is in the orthographic input, rather than in the phonological output (see Toledano & Friedmann, 2023, for transpositions originating in orthographic input vs. phonological output).

In this study, we thus show how dyslexia can be assessed in sign language users, using reading tasks that do not require spoken output, such as reading-then-signing and word–picture matching. Using these tasks, we are able to identify a selective type of dyslexia in signers.

The current study aims to provide a detailed description of how LPD is manifested in the reading of fingerspelling, in order to address questions concerning both the nature of LPD and the cognitive mechanisms underlying finger-reading. The study also aims to demonstrate how selective types of dyslexia can be assessed in deaf signers. Identifying specific types of dyslexia in signers may enable better diagnosis of such impairments in this population and lead to better treatment and teaching for signers with reading impairments.

## 2. General Method

### 2.1. Participants

#### 2.1.1. Participants with Dyslexia

The participants with LPD were two adults who were congenitally deaf late signers of ISL (born to hearing parents and started acquiring sign language during late childhood). Both signers reported difficulties in reading.

##### Alon

Alon was tested in 3 sessions between 2019 and 2021 starting when he was 21 years old. He is a male, born in Israel. In early childhood he underwent several cochlear implant surgeries, which were unsuccessful. This led to him, together with his family, switching to learning Israeli sign language, when he was 3 years old. He graduated from high school. He was not using hearing aids in any of the sessions.

##### Yona

Yona was tested in 4 sessions between 2018 and 2022 starting when she was 20 years old. She is a female, born in Russia, and moved to Israel at the age of 3, where she also started learning sign language, ISL. She is not familiar with any sign languages other than ISL. She used hearing aids from kindergarten until the age of 21; however, she stopped using them because they did not improve her hearing and, as she described, she could only hear noise.

#### 2.1.2. Control Group

The performance of Alon and Yona on the various tasks was compared to control groups of deaf readers of Hebrew and ISL fingerspelling, including native and late signers of ISL, of up to 40 participants depending on the test (total 58 signers; ages 18–60; M = 36.1; SD = 10.4), all of whom use ISL as their main means of communication and are part of the Deaf community in Israel. We specify the size of the control group used in the sections describing each test.

### 2.2. Statistical Analysis

In line with the long tradition of cognitive neuropsychology (Caramazza, 1986; Coltheart, 2006b; Ellis & Young, 1996; Marshall & Newcombe, 1984; Medina & Fischer-Baum, 2017; Nickels et al., 2022; Shallice, 1979, 1988), this study used a single-case approach to describe in detail two cases of signers with dyslexia. We therefore used the statistical methodology accepted for single-case studies (Crawford & Howell, 1998; Crawford & Garthwaite, 2002, 2005, 2007; Crawford et al., 2003, 2011; Willmes, 1990, 2010). This single-case methodology allowed us to compare the performance of each case to the performance of a control group and to determine for each task, stimulus type or error type whether the participant’s performance was significantly different or worse than that of the control participants. We compared the performance of each of the signers with dyslexia to the controls using Crawford and Howell’s (1998) t-test, specifically designed for case–control comparison in single-case studies. This test was shown to minimize type-1 errors compared to other methods (Crawford & Garthwaite, 2012). We used *p* < 0.05. When other tests were used, we specified them in the relevant section.

## 3. Assessment—Method and Results

To test the nature of Alon and Yona’s reading difficulties, we examined their reading of written (printed) words as well as their reading of fingerspelled words sensitive to specific types of dyslexia.

### 3.1. Reading Written Words

Because reading aloud is not an ideal method for assessing reading in deaf signers—due to potential output inaccuracies related to hearing loss rather than true reading difficulties—we used alternative reading tasks: reading-then-signing and word–picture matching.

#### 3.1.1. Reading-Then-Signing Written Words Sensitive to Various Types of Dyslexia

##### Method

We assessed reading-then-signing of Hebrew words sensitive to different types of dyslexia using the existing word-reading screening task from the *TILTAN* battery for the diagnosis of dyslexia in Hebrew (Friedmann & Gvion, 2003), which we adapted to ISL (Friedmann et al., 2021; Haluts et al., 2025). In adapting the test to ISL, we selected words that are familiar to deaf signers, have a sign in ISL, and for which different types of errors lead to a visible change in the sign—namely, for which different errors in the same item would yield different signs from each other and a different sign from the target sign. The participants were presented with 90 words, printed one under the other on a sheet, and were asked to sign the corresponding ISL sign for each word. The items included words sensitive to different known types of dyslexia, including, among others, surface dyslexia, vowel letter dyslexia, neglect dyslexia, visual dyslexia, and attentional dyslexia. Careful analysis of the error pattern in reading can also point to other suspected types of dyslexia (see Friedmann & Coltheart, 2018). Importantly, for the current study, one of the word types that this screening test included was migratable words, which are the stimulus that is most difficult for individuals with LPD, and the test is therefore also sensitive to the identification of LPD. Across all reading tasks, when the participant said that they did not know a given target Hebrew word, this was counted as a “don’t know” response, and reading errors were not counted for this word.

##### Results

**Figure 3:**
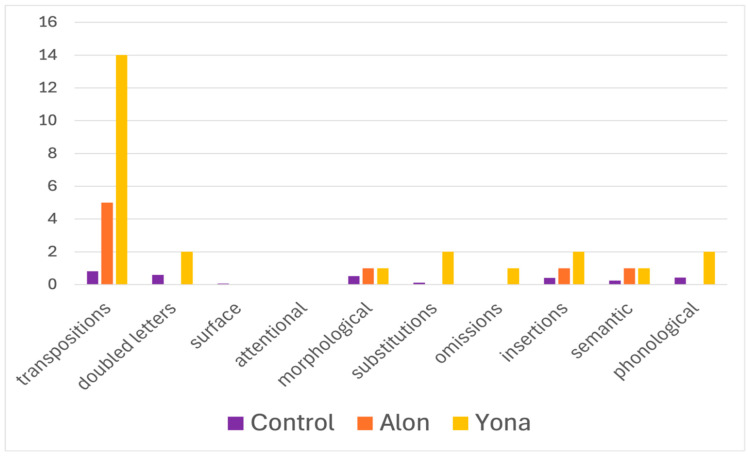
Number of errors of each type that Alon and Yona made on the written screening task, compared to the controls.

Alon and Yona’s most prominent error type in this task was typical of LPD—letter transpositions (mean control = 0.82, SD = 1.19; Alon = 5, t(16) = 3.41, *p* = 0.002 for the comparison of his errors to the controls’; Yona = 14, t(16) = 10.76, *p* << 0.0001). Namely, they signed the sign corresponding to a transposition result of the target word they were reading. For example, for the target word כבלים, KVLIM, cables, they signed dogs, parallel to the word resulting from a transposition of the second and third letters, כלבים, KLVIM. These transpositions occurred exclusively with migratable words—in which a transposition leads to other existing words (including transpositions the participants performed when fingerspelling the target word prior to or instead of signing the target sign, which they sometimes did when they did not understand/did not know the sign, and transpositions in the pronunciation of the Hebrew word that sometimes accompanied their signing). This was by far the most pronounced error type, and none of the other error types exceeded two errors per participant (see Figure 3).

Alon and Yona’s pattern of errors on the screening task led us to suspect they may have letter position dyslexia, a deficit in the encoding of letter position within the word. Thus, we continued with additional tasks of written word reading that are specifically designed to test reading of migratable words.

#### 3.1.2. Reading-Then-Signing Written Migratable Words

##### Method

We further assessed the participants’ letter position encoding by asking them to read and then sign written migratable words, which are the type of stimulus most sensitive to identifying letter position dyslexia (LPD) (Friedmann & Gvion, 2001). We used a migratable words reading test from the TILTAN battery, adjusted to ISL (Friedmann et al., 2021; Haluts et al., 2025).

The participants were presented with 162 migratable words, printed one under the other on a sheet, and were asked to read each word and then sign the corresponding ISL sign.

##### Results

Alon and Yona’s most prominent error type in this task was, again, letter transpositions (in similar rates for consonants and vowels) and errors with doubled letters (see Table 1, which details all relevant error types that exceeded 2% of the target words). In this word list, too, they made significantly more errors of these types than the controls (transpositions: control M = 2%, SD = 1%; Alon = 8%, t(30) = 5.91, *p* << 0.0001; Yona = 15%, t(30) = 12.80, *p* << 0.0001; doubled letters: control M = 1%, SD = 1; Alon = 5%, t(30) = 3.94, *p* = 0.0002; Yona = 4%, t(30) = 2.95, *p* = 0.003).

#### 3.1.3. Comprehension of Written Migratable Words—Word-to-Picture Matching

To further examine Alon and Yona’s transpositions in written word reading, we administered a comprehension task with migratable words.

##### Method

Participants were presented with a written migratable word that appeared in the center of a computer screen for 1200 ms. Then two pictures appeared on the screen and the participants were asked to choose the picture that matched the word by clicking on the corresponding key on the keyboard (“a” for the left picture and “l” for the right picture). The pictures appeared on the screen until a choice was made. The tasks were administered using the Testable platform (see Rezlescu et al., 2020). The words were migratable words, and each pair of pictures included the target word and the result of a letter migration (e.g., the written word “files” followed by pictures of files and flies). The task included a total of 69 items, of which 2 were example items with pairs including the target and a picture of an orthographically unrelated item, 57 test items with migratable pairs, and 10 were fillers with pairs including the target and the result of a letter substitution (e.g., the word “cat” followed by pictures of a cat and a mat). The letter-substitution fillers were included to allow for a comparison between the letter-migration pairs and pairs with a similar Levenshtein distance (Navarro, 2001).

##### Results

Yona’s performance with migratable words was significantly worse than the controls’ (64% correct, controls: mean = 97%, SD = 4%, t(28) = 8.11, *p* << 0.0001)^1^. In contrast, she made only one error with the letter-substitution fillers. Alon’s accuracy rate was comparable to the controls both with migratable words (96% correct) and with fillers (100%).

Both Alon and Yona had significantly longer reaction times (RTs) for the migratable test items than the controls^2^ (mean controls: 1367 ms, SD = 358; Alon: mean = 3068 ms, t(28) = 4.67, *p* << 0.0001; Yona: mean = 3700 ms, t(28) = 6.41, *p* << 0.0001). Interestingly, the difference between mean RTs for migratable test items and letter-substitution fillers was significantly greater for both Alon and Yona than for the controls (control mean difference: 116 ms, SD = 281; Alon: 930, t(28) = 2.85, *p* < 0.01; Yona: 905, t(28) = 2.76, *p* < 0.01), indicating a greater cost of the need to accurately analyze letter position within the word for both participants with impairments than for the controls.

### 3.2. Reading Fingerspelled Words

The tests of reading written words indicated that Alon and Yona had an impairment in letter position encoding. We next examined their reading of fingerspelled words, which is an integral process in the use of sign languages including ISL, to answer two theoretical questions. First, it was investigated whether written and fingerspelled words are read via shared cognitive mechanisms. If we found that LPD affects written and fingerspelled words in a similar way and leads to similar pattern of errors, we can conclude that both modes of presentation are processed by shared mechanisms and that the deficit leading to LPD affects these shared mechanisms.

A second question that can be answered by testing finger-reading in individuals with LPD is whether LPD is a visual deficit which stems from the simultaneous presentation of the letters in the word. One possible explanation for the pattern of errors seen in individuals with LPD is that a deficit in visually separating the letters is the factor causing difficulty with encoding their position (as has been suggested for dyslexia in general by approaches relating to effects such as crowding, Gori & Facoetti, 2015; Martelli et al., 2009; Spinelli et al., 2002). Because the fingerspelled letters do not appear simultaneously in one visual presentation but rather are signed one after the other with a temporal distinction between them, if LPD is indeed a deficit in visual separation, we would expect the letter-by-letter presentation to remove interference from neighboring letters, leading to a reduction in transposition errors when the words are fingerspelled. Alternatively, if LPD is an impairment in orthographic processing of letter position rather than a visuo-spatial impairment (as claimed by, e.g., Friedmann & Gvion, 2001; Friedmann & Haddad-Hanna, 2012; Friedmann & Rahamim, 2007; Güven & Friedmann, 2019; Kohnen et al., 2012; Potier Watkins et al., 2023; and Traficante et al., 2024), we would expect a similar pattern of errors regardless of presentation modality.

To test these two questions, we administered the same tests described in Section 3.1 to Alon and Yona, this time with *fingerspelled* words.

#### 3.2.1. Reading-Then-Signing Fingerspelled Words Sensitive to Various Types of Dyslexia

##### Method

Participants watched a video in which a native signer fingerspelled a word at a slow but natural pace. The participants were asked to sign the corresponding ISL sign (e.g., they saw B-O-A-T fingerspelled and had to sign boat). The words were similar to the items in the written words reading task, described in Section 3.1.1, but in a mixed order. Alon and Yona read 81 and 66 items in this test, respectively, and their performance was compared to that of the controls for the same items.

##### Results

**Figure 4:**
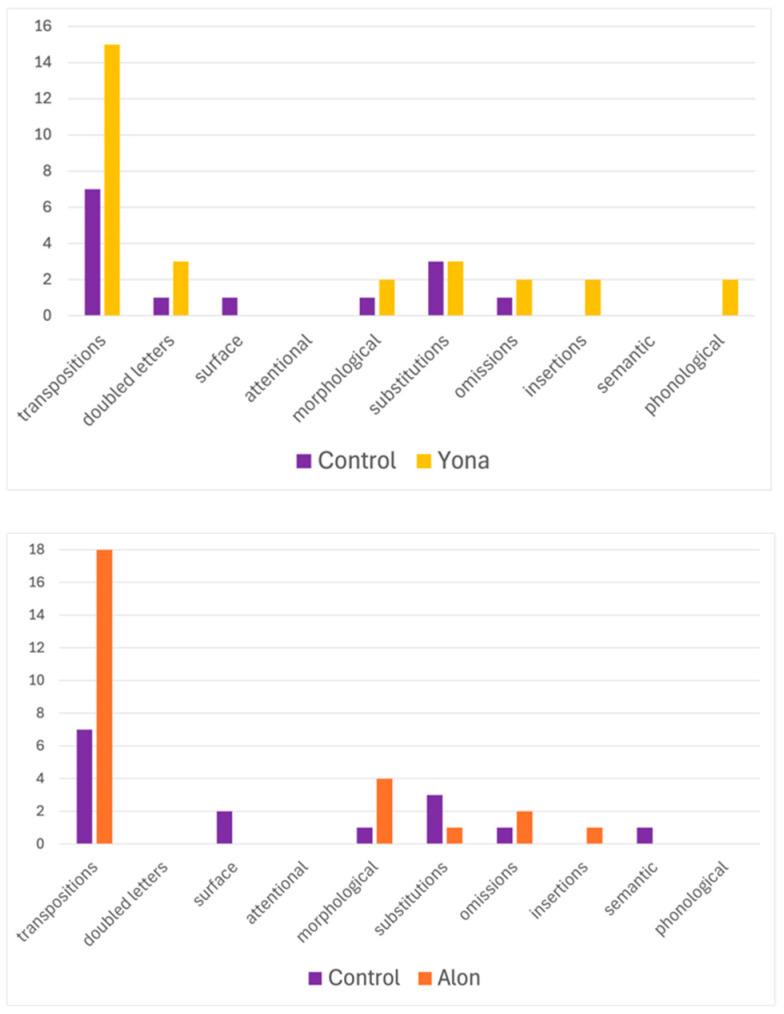
Percentage of errors of each type that Alon and Yona made on the fingerspelled screening task, compared to the controls.

In their reading of fingerspelled words, similarly to their reading of written words, Alon and Yona made mainly letter transpositions. Alon made transpositions on 19% of the words he read, significantly more transpositions than the control group for the items he read (control M = 7%, SD = 6%, t(7) = 1.89, and *p* = 0.049). Yona had 15% transpositions, trending towards but not significantly greater than the controls for the items she read (control M = 7%, SD = 5%, t(7) = 1.52, and *p* = 0.08). As in the written word version of this test (Section 3.1.1), these transpositions occurred exclusively in migratable words. Alon also made three morphological errors (Figure 4). Yona said she did not know the word/sign for 20 of the items (30%), possibly a reflection of her limited Hebrew lexicon. Other error types did not exceed two errors. Thus, in a test that was designed to expose all kinds of dyslexias, via the elicitation of all kinds of reading errors, the participants showed a picture of a selective deficit in reading which affected the encoding of letter position, just like when reading written words.

#### 3.2.2. Reading-Then-Signing Fingerspelled Migratable Words

##### Method

Participants watched a video of a native signer fingerspelling a word at a slow but natural pace and were then asked to sign the corresponding ISL sign. The words were the same migratable words that appeared as written words in the task described in Section 3.1.2 but in a different order. Alon performed the full test (162 items), and Yona finger-read 100 of the items, as did the controls.

##### Results

Alon and Yona’s most prominent error type in the fingerspelled word reading task was, like in their reading of the written words, letter transpositions. They performed significantly more transpositions than the controls (Alon = 38%, t(12) = 4.98, *p* = 0.0002; Yona = 25%, t(12) = 3.37, *p* = 0.003). They also made significantly more errors with doubled letters than the controls (Alon 6%, t(12) = 2.41, *p* = 0.02; Yona 4%, t(12) = 1.45, *p* = 0.09). See Table 2.

Interestingly, for many of the words he read (24 words), Alon tried to fingerspell the target word himself, before producing the sign or when his error in reading did not lead to an existing word. In these self-initiated fingerspelled responses, he also performed mostly middle-letter migrations (9) and errors with doubled letters (3). These errors were almost always compatible with his transposition error reflected in the sign he produced (e.g., instead of KVLIM, cables, he signed the result of a transposition, KLVIM, dogs, and made the same error when he fingerspelled the word to himself, which he fingerspelled K-L-V-I-M).

Alon and Yona also made some morphological errors in this test (M controls = 2%, SD = 1%; Alon = 8%, t(12) = 5.78, *p* < 0.0001; Yona = 9%, t(12) = 6.75, *p* < 0.0001). Their letter errors (substitutions, omissions, and insertions not involving doubled letters) did not exceed two errors (with the exception of Yona’s five consonant substitutions). Yona said she did not know the word/sign for 26 of the items in this test.

#### 3.2.3. Comprehension of Fingerspelled Migratable Words—Word-to-Picture Matching

##### Method

Participants watched a video in which a native signer fingerspelled a word at a slow but natural pace^3^. The video appeared in the center of the screen until the word was completed, followed by two pictures. The participants were then asked to choose the picture matching the word, in a similar procedure to the one described in Section 3.1.3 above, with the same test items in a mixed order and with different example items and fillers.

##### Results

Both Alon and Yona had a significantly worse performance for migratable words than the controls (M controls = 94%, SD = 5%; Alon = 82%, t(21) = 2.35, *p* = 0.03; Yona = 53%, t(21) = 8.02, *p* << 0.0001). Alon also made two errors on the letter-substitution fillers.

As in the written version of the test described above in Section 3.1.3, Alon and Yona had significantly longer RTs for the migratable test items than the controls (M controls = 1074 ms, SD = 418; Alon = 2131 ms, t(21) = 2.47, *p* = 0.02; Yona = 2378 ms, t(21) = 3.05, *p* = 0.006)^4^.

#### 3.2.4. Interim Summary—Similar Pattern When Reading Written and Fingerspelled Words

The results of the reading tasks presented so far show that Alon and Yona had very similar patterns of errors when reading written and fingerspelled words. Their reading of written words showed mostly errors typical of LPD: letter transpositions and errors in doubled letters, both when reading-then-signing and in a picture-matching task. This resembles the error pattern of hearing speakers with such an impairment (Friedmann & Gvion, 2001; Friedmann & Haddad-Hanna, 2012; Friedmann & Rahamim, 2007, 2014; Güven & Friedmann, 2019; Kohnen et al., 2012; Potier Watkins et al., 2023; Traficante et al., 2024).

Strikingly, their finger-reading showed exactly the same pattern of errors, with mostly letter transpositions and errors with doubled letters, demonstrating for the first time that LPD is manifested similarly for written and fingerspelled words.

To test whether letter transpositions occur even when comprehension of the word is not required (and hence, to rule out the semantic component as a source for the transpositions), we administered tasks of repetition and lexical decision of fingerspelled words.

#### 3.2.5. Repetition of Fingerspelled Words

##### Method

Participants watched a video of a native signer fingerspelling a word at a slow but natural pace and were asked to repeat the fingerspelling (i.e., to fingerspell the word themselves). The test included 25 migratable words of 4–6 letters (M = 4.8, SD = 0.8) and one training item of 3 letters, with which no participant made any errors.

##### Results

Alon and Yona’s most prominent errors in this task were letter transpositions. They made significantly more transpositions than the controls (M controls = 6%, SD = 7%; Alon = 36%, t(21) = 4.19, *p* = 0.0002; Yona = 24%, t(21) = 2.51, *p* = 0.01), and Yona also made significantly more errors with doubled letters (M controls = 2%, SD = 4%; Yona = 12%, t(21) = 2.45, *p* = 0.01). Alon also made three consonant substitutions. Neither of the participants committed any additional errors in this task.

#### 3.2.6. Lexical Decision of Fingerspelled Words

##### Method

Participants were presented with a fingerspelled Hebrew word or pseudo-word and were requested to decide whether it was a real word (by responding “yes” or “no”). The stimuli were 41 letter sequences: 16 migratable words, 20 migratable pseudo-words (in which a transposition creates an existing word), and 5 non-migratable pseudo-words (in which a transposition does not create an existing word. Yona was not presented with these 5 items).

##### Results

Both Alon and Yona performed worse than the controls with migratable words (i.e., they judged the existing migratable word as nonexistent; M controls = 2% errors, SD = 3%; Alon = 13%, t(10) = 3.51, *p* = 0.003; Yona = 19%, t(10) = 5.43, *p* = 0.0001). With migratable pseudo-words, the controls also performed rather poorly (i.e., responded that the pseudo-word was a real word; M = 28% errors, SD = 17%); however, Alon still performed much worse (75% errors, t(10) = 2.65, *p* = 0.01). In contrast, Alon did not make any errors with the five non-migratable pseudo-words.

#### 3.2.7. Interim Summary

The performance of Alon and Yona for repetition and lexical decision of fingerspelled words further suggests that their impairment does not stem from a comprehension/semantic-related deficit: even in such tasks where reaching the meaning of the word is not necessary for performance, both participants still performed many letter transpositions and showed difficulty with migratable words and pseudo-words.

#### 3.2.8. Letter-by-Letter Reading

Fingerspelled words differ from written words in that they are signed letter by letter. Nevertheless, we found that both signers with LPD performed transpositions in fingerspelled words. We further tested reading of letters that appear sequentially by presenting the *written* words letter by letter, at the same location on the screen with temporal separation between them. Only Yona performed this task, as we came up with this test rather late in the evolution of this study, when Alon was no longer available for testing.

##### Method

We presented Yona with 20 migratable written words (and three fillers) presented in such a way that each letter of the word appeared in the center of the screen for 750 ms, followed by a question mark. When the question mark appeared, the participant was requested to sign the word. 

##### Results

Yona performed mostly letter transpositions in this task (45%), significantly more than the controls (M controls = 10%, SD = 9%, t(11) = 3.74, *p* = 0.002). She said she did not know the word/sign for four of the words.

The fact that Yona made similar transposition errors when the written word was presented letter by letter, with temporal rather than spatial separation between the letters, suggests that LPD cannot be explained by a deficit in visually separating between simultaneously presented letters.

### 3.3. Pattern of Letter Migrations—Middle- Versus Exterior-Letter Transpositions

Previous studies have shown that speakers of spoken languages with LPD, when reading written words, perform letter transpositions mostly with letters in the middle of the word. Transpositions involving an exterior letter of the word (i.e., the first or final letters) occur at a far lower rate (Friedmann & Rahamim, 2007; Kohnen et al., 2012).

In all the reading-then-signing tasks, we examined whether Alon and Yona also showed the same pattern of mainly middle-letter migrations in fingerspelling (and whether their written word reading replicated earlier findings regarding middle-letter transposition being the most common).

We analyzed all target words in which both middle- and exterior-letter migrations could create other existing words, in all of the tests with signed/fingerspelled output. This analysis indicated that the participants with LPD performed many middle-letter migrations with these words and only a few exterior migrations. Alon made 42 middle migrations and 5 exterior migrations; Yona made 41 middle migrations and 2 exterior migrations. For both participants, the number of middle-letter transpositions was significantly greater than the number of exterior-letter transpositions, both for written words (χ^2^ = 8.99, *p* = 0.003 for both participants) and for fingerspelled words (Alon: χ^2^ = 21.79, *p* < 0.00001; Yona: χ^2^ = 14.52, *p* = 0.0001).

We also compared the middle and exterior migrations to the control group. We examined their middle- and exterior-letter transposition rate when reading-then-signing written migratable words (Section 3.1.2) and when reading-then-signing fingerspelled migratable words (Section 3.2.2). For written words, both Alon and Yona performed significantly more middle-letter transpositions than the controls (control M = 1%, SD = 1%; Alon = 8%, t(30) = 6.89, *p* << 0.0001; Yona = 14%, t(30) = 12.79, *p* << 0.0001). Importantly, their rate of exterior-letter transpositions was comparable to that of the controls (control M = 0%, SD = 0%; Alon = 0%; Yona = 0.6%). A strikingly similar pattern was observed in finger-reading. When reading-then-signing fingerspelled words, both participants also performed significantly more middle-letter transpositions than the controls (M = 7%, SD = 5%; Alon = 34%, t(12) = 5.20, *p* = 0.0001, Yona = 25%, t(12) = 3.47, *p* = 0.002). Again, their exterior-letter transpositions were comparable to those of the controls (Yona = 0%; M controls = 0%, SD = 1%) or at a much-lower rate than middle-letter transpositions (Alon = 3%).

### 3.4. Assessing Additional Skills—Intact Processes

#### 3.4.1. Reading-Then-Signing Multi-Digit Numbers

If the letter-position errors made by Alon and Yona are due to a general visuo-spatial deficit, we would expect that not only words but also other types of visually presented stimuli that are sequences of symbols, such as numbers, would show transpositions. To test whether reading numbers leads to similar errors, we administered a task of reading-then-signing multi-digit numbers.

##### Method

We presented the participants with 60 multi-digit numbers of three to six digits, printed on a sheet one under the other (MAYIM battery, Dotan & Friedmann, 2014; see also Friedmann et al., 2021, and Haluts et al., 2025). We requested the participants to read each number and sign it in ISL.

##### Results

Alon and Yona performed comparable to the controls when reading-then-signing numbers (Alon = 83% correct; Yona = 90%; M controls = 88%, SD = 8%, N = 24). Specifically, Alon and Yona did not make any transposition errors in this task.

The performance of Alon and Yona for reading-then-signing multi-digit numbers shows a sharp contrast with their performance for reading-then-signing written and fingerspelled words. Although they make mostly letter-position errors and errors with doubled letters when reading words, as expected in LPD, they make absolutely no order errors when reading numbers. This shows a dissociation between the cognitive processes underlying word and number reading, in line with previous findings from hearing speakers with LPD and with dysnumeria (Dotan & Friedmann, 2019). It also rules out a general visual account for their transposition errors in reading words.

#### 3.4.2. Serial Recall Tests

If the letter-position errors are related to impaired phonological working memory (pWM), we would expect both participants with impairment to perform poorly on pWM tasks. To assess participants’ pWM, we used serial recall tasks of existing signs and of pseudo-signs, in which the participants had to see, hold in memory, and repeat sequences of signs (from the SIMBA test battery for working memory in ISL, Haluts & Friedmann, 2019; see Haluts & Friedmann, 2026, in press).

##### Method

Participants were presented with sets of signs increasing in length from 2 to 5 signs in each set, with five sets for each length. The span score for each participant was determined by the maximum length (i.e., number of items) for which they could correctly recall at least three sets. For two correct sets for a given length, we added 0.5 points to their span score (e.g., Alon could repeat three sets of three items and two sets of four items, so his span was 3.5).

##### Results

Both Alon and Yona had spans comparable to the controls for pseudo-signs (Alon = 3.5; Yona = 2; M controls = 2.44, SD = 0.50, N = 36). In the existing signs span test, Alon showed a span comparable to the controls (Alon = 5; control span M = 4.26, SD = 0.62), and Yona showed a span that was smaller than that of the controls (Yona = 3, t(39) = 2.01, *p* = 0.026).

Alon’s intact (and even relatively high) performance on serial recall suggests that his errors with letter position in finger-reading cannot be attributed to a pWM deficit, suggesting another dissociation between phonological processing of signs and pseudo-signs and the processing of fingerspelling.

## 4. Discussion

This study reports two cases of deaf signers with letter position dyslexia for both written and fingerspelled words. It forms the first report of a selective type of dyslexia in deaf signers and the first neuropsychological study to investigate specific types of dyslexia in this population. It is also the first to explore the way a selective type of dyslexia is manifested in the reading of fingerspelling, comparing the way the same type of dyslexia is manifested in fingerspelled and written words.

We administered a comprehensive battery of reading tasks to two deaf signers who reported difficulties in reading. Their pattern of errors when reading-then-signing written words sensitive to various types of dyslexia indicated a selective letter position dyslexia, a deficit in letter position encoding at the stage of orthographic-visual analysis (Friedmann & Gvion, 2001; Friedmann & Haddad-Hanna, 2012; Friedmann & Rahamim, 2007, 2014; Güven & Friedmann, 2019; Kohnen et al., 2012; Potier Watkins et al., 2023; Traficante et al., 2024). Their errors included mainly within-word transpositions with almost no errors of other types. Their letter position dyslexia manifested not only in reading-then-signing but also in tasks of migratable word–picture matching, in which they showed lower accuracy (for Yona) and longer RTs (for both participants) for migratable words, as well as in a lexical decision task of migratable nonwords and in fingerspelling repetition (finger-read and then fingerspell).

### 4.1. Similar Patterns of Impairment in Reading Written and Fingerspelled Words

Previous research of LPD in the reading of written words has yielded several central characteristics in a variety of tasks, including reading aloud, word–picture matching, and lexical decision tasks. Transpositions occur mostly with *middle letters* and very rarely involve exterior letters (Friedmann & Gvion, 2001; Friedmann & Rahamim, 2007); in addition to transpositions, individuals with LPD also make errors of omission for one instance of a *doubled letter*, i.e., a letter that appears twice in the word, and also errors of doubling letters (Friedmann & Rahamim, 2007); errors occur mainly in *migratable words* and far less often in non-migratable words (Friedmann & Rahamim, 2007; Kohnen et al., 2012). These characteristics were found in hearing participants with LPD in various spoken languages (Friedmann & Gvion, 2001; Friedmann & Haddad-Hanna, 2012; Friedmann & Rahamim, 2007; Güven & Friedmann, 2019; Kohnen et al., 2012; Potier Watkins et al., 2023; Toledano & Friedmann, 2023; Traficante et al., 2024). The pattern of errors that Yona and Alon, the deaf signing participants, showed in the reading of written words is perfectly in line with these characteristics. They show transpositions mainly involving middle letters but very few migrations involving exterior letters; they show errors with doubled letters; and their errors occur mainly in migratable words.

Importantly, both signers with LPD showed a strikingly similar pattern in finger-reading. When reading fingerspelled words, like when reading written words, they performed mostly middle-letter migrations, with very few exterior-letter migrations; they made errors with doubled letters; and they made errors mainly in migratable words. This pattern was present both in reading-then-signing and in the fingerspelled version of the word–picture matching. The pattern was also present in tasks that did not involve comprehension—lexical decision and repetition of fingerspelled words and pseudo-words.

The fact that the same type of dyslexia was present in the reading of written words and in the reading of fingerspelled words, together with the finding that the characteristics of this dyslexia were the same when reading written and fingerspelled words, provide strong evidence that the reading of fingerspelling uses the same cognitive mechanisms as reading written words in deaf signers.

### 4.2. Where in the Neuropsychological Model of Reading Is Their Deficit?

Where exactly in the neuropsychological model of reading is their deficit? We suggest that it is, like previously described cases of LPD, in the orthographic-visual analyzer, in the function of letter position encoding.

A deficit in an earlier, general visuo-spatial component is ruled out based on their good reading of multi-digit numbers, which was at the same level of performance as the controls. This suggests that their deficit lies in a stage that is already orthographic-specific.

Their pattern of errors also rules out a deficit at a stage that follows the orthographic-visual analyzer, the orthographic input buffer (OIB). The OIB holds the output of all the functions of the orthographic-visual analyzer, including letter identity, letter position within the word, and letter–word binding. Hence, individuals with this type of impairment make errors related to all these functions, including errors of letter identity, errors of letter position including the position of exterior letters, and letter migrations between words (Friedmann & Coltheart, 2018; Sternberg & Friedmann, 2007, 2009). However, Alon and Yona showed a very selective deficit, which affected only the position of middle letters. They did not make other errors that are typical for an OIB deficit: they did not make letter substitutions, additions, and omissions (when the letter was not doubled)^5^. They performed only very few within-word transpositions that involved exterior letters. They also did not perform migrations of letters between words, which are very common in OIB deficits.

Another alternative explanation that is readily ruled out is that Alon and Yona’s errors are simply a reflection of a lexical strategy whereby they are looking, in their vocabulary, for a close-enough word that they know, and this is what drives their letter position errors. This alternative explanation would suggest that when they do not have the target word in their orthographic lexicon, or when they decode the letter sequence only inaccurately, they then select an existing word that matches a large enough part of the encoded letter string. This hypothesis is completely untenable given the results. Had this been the case, we would also expect letter omissions, additions, and substitutions, as well as exterior-letter migrations. We did not see almost any of these errors, as the participants made middle-letter migrations almost exclusively. We would also expect errors in non-migratable words, which were also not witnessed. Thus, an alternative account, whereby the errors arise solely from approximate decoding combined with reliance on existing vocabulary, can be completely discounted.

To conclude, we suggest that Alon and Yona have a selective deficit in the orthographic-visual analyzer, in the function that encodes letter positions. This function receives the representation of the ordered abstract letter identities together and then analyzes the relative letter position of each letter, be it written or fingerspelled. A possible model extension would be a fingerspelling buffer that holds the sequentially presented letters together and provides the orthographic-visual analyzer with the entire sequence of letters for the encoding of letter order.^6^

### 4.3. From LPD in Fingerspelling to the Nature of the Impairment in LPD

Testing signers with LPD enabled us to answer theoretical questions regarding the nature of this impairment. Because fingerspelling in the surrounding spoken language alphabet is very common for various items that do not have a sign, such as names, finger-reading is also very natural for signers. This allowed us to examine how LPD is manifested when reading words spelled one letter at a time with temporal rather than visuo-spatial separation between the letters.

The fact that signers with LPD still made transposition errors when they read fingerspelled words, even though letters in fingerspelled words are visually separately presented, suggests that LPD is not a general difficulty in the visuo-spatial separation of the letters. For example, some researchers suggested that dyslexia is related to crowding, a deficit that affects visuo-spatial separation, which impairs the ability to recognize objects in clutter (Gori & Facoetti, 2015; Martelli et al., 2009; Spinelli et al., 2002). Although it might be that there is a subset of readers who do experience increased crowding (Joo et al., 2018), crowding cannot underlie LPD, given that in this study, the individuals with LPD also showed the same effects when letters were presented sequentially, one by one. Rather, LPD seems to emerge from an orthographic deficit in letter position encoding.

Additionally, in tasks of reading aloud in spoken languages, there is a chance that transpositions actually result from a deficit in the phonological output, rather than a frank deficit in the orthographic-visual analyzer (Toledano & Friedmann, 2023). Namely, an error of reading “slime” as “smile” can either emerge in the orthographic input stage or in the phonological production stage. In reading-then-signing tasks, this is not an issue, and a transposition can be confidently ascribed to orthographic input. The signs for “smile” and “slime” are very different phonologically, so signing one instead of the other cannot be ascribed to a failure in the phonological output stage. Or, to give an example from Hebrew and ISL, the two Hebrew migratable words כבלים (KVLIM, “cables”) and כלבים (KLVIM, “dogs”) differ only in the relative position of two middle letters, but their corresponding signs are very different with respect to handshape and movement. Therefore, signing one sign instead of the other cannot result from a phonological error but rather only from an orthographic input deficit.

### 4.4. Dissociations with Theoretical Bearing

Our results also show, for the first time in deaf signers, theoretically important dissociations between different cognitive processes. Alon and Yona performed many letter transpositions when reading-then-signing words; however, they did not perform any digit transpositions when reading-then-signing multi-digit numbers. Together with our previous findings of a deaf signer with dysnumeria who showed errors when reading-then-signing numbers but had a completely intact ability to read words (Friedmann et al., 2021), this points to a double dissociation between reading words and reading numbers in deaf signers. This indicates that separate cognitive mechanisms participate in these processes (as previously shown for hearing speakers, Friedmann et al., 2010b; Güven & Friedmann, 2019; Hannagan et al., 2015; Shum et al., 2013; see Dotan & Friedmann, 2019, for a review).

We also show a dissociation between LPD and an impairment in phonological WM (pWM). Alon had an intact, and even slightly high, performance with serial recall of existing signs and pseudo-signs, suggesting that his errors when reading, and most importantly when reading sequentially presented fingerspelled letters, cannot be explained by an impaired pWM.

### 4.5. Methodological Considerations for the Diagnosis of Dyslexia Types in Deaf Signers

This study proposes a methodology for identifying distinct types of dyslexia in deaf signers. Because reading aloud is not an ideal method for assessing reading in this population—due to potential output inaccuracies related to hearing loss rather than true reading difficulties—alternative tasks are recommended. These tasks include reading-then-signing, word–picture matching, lexical decision tasks, and finger-reading-then-fingerspelling (i.e., repetition of fingerspelling).

Given that the written language is often not the native language of many deaf signers nor their main language of communication, test items should be familiar enough for this population. Additionally, it is essential to ensure that both the target word and potential error responses have clear, recognizable signs in the relevant sign language and that these signs are distinct enough—from the target word and from one another—to make errors observable through signing.

To accurately map the impairment to a specific component in the neuropsychological model, it is crucial to incorporate reading tasks that include both written and fingerspelled words and to compare error patterns across these two modes of presentation.

To identify the whole spectrum of dyslexia types (Friedmann & Coltheart, 2018), the tasks have to include target words that are sensitive to the detection of all known types of dyslexia (migratable words, word pairs that allow for letter migration between them, nonwords, irregular and unpredictable words, morphologically complex words, long words, function words, and more).

## 5. Conclusions

This study shows, for the first time, the manifestation of letter position dyslexia in fingerspelling. It has theoretical-neuropsychological contributions regarding the nature of this impairment and the cognitive systems involved in reading in deaf signers: it shows that LPD is an impairment in the encoding of letter position rather than general visuo-spatial analysis. It also shows that the cognitive mechanisms underlying the reading process are shared by hearing speakers and deaf signers and for reading written and fingerspelled words. The study also has clinical and methodological contributions by demonstrating how selective reading impairments can be identified in deaf signers, which may enable better diagnosis and better treatment of dyslexia in deaf signers, when they read written words and when they read fingerspelled words.

## Figures and Tables

**Table 1:** Percentage of errors of different types made by Alon, Yona, and the controls (average and SD) when reading-then-signing written migratable words.

	Control	Alon	Yona
Letter transpositions	2 (2)	8	15
Doubled letter error	1 (1)	5	4
Morphological	1 (1)	6	6
Don’t know	1 (2)	3	27

**Table 2:** Percentage of errors (and SD) of different types made by Alon, Yona, and the controls when reading-then-signing fingerspelled migratable words.

	Control	Alon	Yona
Letter transpositions	7 (6)	38	25
Doubled letter error	1 (2)	6	4
Morphological	2 (1)	8	9
Consonant substitutions	0.8 (1.4)	2	5
Don’t know	1 (2)	3	26

## Data Availability

The original contributions presented in this study are included in the article. Further inquiries can be directed to the corresponding author.

## Notes

1 In this task as well as the parallel task for finger-reading (Section 3.2.3), we excluded outliers—items in which a participant had RTs longer than 3 SD of their mean RT—which resulted in the exclusion of 0–3 items for each participant (written version: mean = 1.47 items, SD = 0.73; fingerspelled version: mean = 1.64, SD = 0.70).

2 For the analysis involving RTs, we only used items for which the participants responded correctly. Taking into account all of the items leads to similar patterns.

3 The fingerspelling was presented in a video (rather than by live signing) to ensure all the participants were tested with the exact same stimuli. However, if a participant had difficulty understanding a specific item in the video, the native signer experimenter was present in the room and could fingerspell it to them.

4 On this test, RTs are harder to interpret, because we calculated them from the moment the video of the fingerspelling disappeared and the two pictures appeared on the screen; however, we assume that orthographic-visual analysis begins during the video presentation.

5 Alon and Yona did make some morphological errors in some reading and signing tasks, in which they signed the correct root but with an incorrect morphology. However, when they saw a written and fingerspelled word and fingerspelled it, they did not make any morphological errors, suggesting that the input orthographic stages did not lead to the morphological errors. We suggest that these morphological errors in signing morphologically complex Hebrew words resulted from Alon and Yona’s unfamiliarity with the target Hebrew words, leading them to sign them according to the root in a morphological template that created a Hebrew word they did know. The fact that Yona often said she did not know the target Hebrew words or their corresponding sign supports this conclusion, as it indicates her relatively small vocabulary, which may lead her to try and comprehend words based on their root.

6 There may be a selective deficit in this input buffer responsible for holding fingerspelled letters together. In this case, we expect a deficit in reading fingerspelling but not in reading written words, and we predict this stage to be sensitive to word length (number of letters).

## References

[B1-behavsci-16-00654] Allen T. E., Schildroth A. N., Karchmer M. A. (1986). Patterns of academic achievement among hearing impaired students: 1974 and 1983. Deaf Children in America.

[B2-behavsci-16-00654] Beal-Alvarez J. S., Lederberg A. R., Easterbrooks S. R. (2012). Grapheme–phoneme acquisition of deaf preschoolers. Journal of Deaf Studies and Deaf Education.

[B3-behavsci-16-00654] Beauvois M. F., Dérouesné J. (1979). Phonological alexia: Three dissociations. Journal of Neurology, Neurosurgery & Psychiatry.

[B4-behavsci-16-00654] Blackburn D. W., Bonvillian J. D., Ashby R. P. (1984). Manual communication as an alternative mode of language instruction for children with severe reading disabilities. Language, Speech, and Hearing Services in Schools.

[B5-behavsci-16-00654] Bolger D. J., Perfetti C. A., Schneider W. (2005). Cross-cultural effect on the brain revisited: Universal structures plus writing system variation. Human Brain Mapping.

[B6-behavsci-16-00654] Bub D., Cancelliere A., Kertesz A., Patterson K. E., Marshall J. C., Coltheart M. (1985). Whole word and analytic translation of spelling to sound in a non-semantic reader. Surface dyslexia: Neuropsychological and cognitive studies of phonological reading.

[B7-behavsci-16-00654] Burden V., Campbell R. (1994). The development of word-coding skills in the born deaf: An experimental study of deaf school-leavers. British Journal of Developmental Psychology.

[B8-behavsci-16-00654] Caramazza A. (1986). On drawing inferences about the structure of normal cognitive systems from the analysis of patterns of impaired performance: The case for single-patient studies. Brain and Cognition.

[B9-behavsci-16-00654] Castles A. (2006). The dual route model and the developmental dyslexia. London Review of Education.

[B10-behavsci-16-00654] Cecchetto C., Pfau R., Steinbach M., Woll B. (2016). The syntax of sign language and universal grammar. Sign language: An international handbook.

[B11-behavsci-16-00654] Cohen L., Dehaene S., Naccache L., Lehéricy S., Dehaene-Lambertz G., Hénaff M. A., Michel F. (2000). The visual word form area: Spatial and temporal characterization of an initial stage of reading in normal subjects and posterior split-brain patients. Brain.

[B12-behavsci-16-00654] Cohen L., Lehéricy S., Chochon F., Lemer C., Rivaud S., Dehaene S. (2002). Language-specific tuning of visual cortex? Functional properties of the Visual Word Form Area. Brain.

[B13-behavsci-16-00654] Coltheart M. (2006a). Dual route and connectionist models of reading: An overview. London Review of Education.

[B14-behavsci-16-00654] Coltheart M. (2006b). John Marshall and the cognitive neuropsychology of reading. Cortex.

[B15-behavsci-16-00654] Coltheart M., Kohnen S., Faust M. (2012). Acquired and developmental disorders of reading and spelling. The handbook of the neuropsychology of language.

[B16-behavsci-16-00654] Coltheart M., Rastle K., Perry C., Langdon R., Ziegler J. (2001). DRC: A dual route cascaded model of visual word recognition and reading aloud. Psychological Review.

[B17-behavsci-16-00654] Crawford J. R., Garthwaite P. H. (2002). Investigation of the single case in neuropsychology: Confidence limits on the abnormality of test scores and test score differences. Neuropsychologia.

[B18-behavsci-16-00654] Crawford J. R., Garthwaite P. H. (2005). Testing for suspected impairments and dissociations in single-case studies in neuropsychology: Evaluation of alternatives using Monte Carlo simulations and revised tests for dissociations. Neuropsychology.

[B19-behavsci-16-00654] Crawford J. R., Garthwaite P. H. (2007). Comparison of a single case to a control or normative sample in neuropsychology: Development of a Bayesian approach. Cognitive Neuropsychology.

[B20-behavsci-16-00654] Crawford J. R., Garthwaite P. H. (2012). Single-case research in neuropsychology: A comparison of five forms of t-test for comparing a case to controls. Cortex.

[B21-behavsci-16-00654] Crawford J. R., Garthwaite P. H., Gray C. D. (2003). Wanted: Fully operational definitions of dissociations in single-case studies. Cortex.

[B22-behavsci-16-00654] Crawford J. R., Garthwaite P. H., Ryan K. (2011). Comparing a single case to a control sample: Testing for neuropsychological deficits and dissociations in the presence of covariates. Cortex.

[B23-behavsci-16-00654] Crawford J. R., Howell D. C. (1998). Comparing an individual’s test score against norms derived from small samples. The Clinical Neuropsychologist.

[B24-behavsci-16-00654] Crume P. K. (2013). Teachers’ perceptions of promoting sign language phonological awareness in an ASL/English bilingual program. The Journal of Deaf Studies and Deaf Education.

[B25-behavsci-16-00654] Delage H., Tuller L. (2007). Language development and mild-to-moderate hearing loss: Does language normalize with age?. Journal of Speech, Language, and Hearing Research.

[B26-behavsci-16-00654] Dotan D., Friedmann N. (2014). MAYIM: Battery for the assessment of mathematical learning disabilities.

[B27-behavsci-16-00654] Dotan D., Friedmann N. (2019). Separate mechanisms for number reading and word reading: Evidence from selective impairments. Cortex.

[B28-behavsci-16-00654] Ellis A. W., Young A. W. (1996). Human cognitive neuropsychology: A textbook with readings.

[B29-behavsci-16-00654] Enns C., Lafond L. D. (2007). Reading against all odds: A pilot study of two deaf students with dyslexia. American Annals of the Deaf.

[B30-behavsci-16-00654] Fischler I. (1985). Word recognition, use of context, and reading skill among deaf college students. Reading Research Quarterly.

[B31-behavsci-16-00654] Friedmann N., Coltheart M., Bar-On A., Ravid D. (2018). Types of developmental dyslexia. Handbook of communication disorders: Theoretical, empirical, and applied linguistic perspectives.

[B32-behavsci-16-00654] Friedmann N., Dotan D., Rahamim E. (2010a). Is the visual analyzer orthographic-specific? Reading words and numbers in letter position dyslexia. Cortex.

[B33-behavsci-16-00654] Friedmann N., Gvion A. (2001). Letter position dyslexia. Cognitive Neuropsychology.

[B34-behavsci-16-00654] Friedmann N., Gvion A. (2003). TILTAN: Battery for the diagnosis of dyslexias in Hebrew.

[B35-behavsci-16-00654] Friedmann N., Haddad-Hanna M. (2012). Letter position dyslexia in Arabic: From form to position. Behavioural Neurology.

[B36-behavsci-16-00654] Friedmann N., Haddad-Hanna M., Saiegh-Haddad E., Joshi M. (2014). Types of developmental dyslexia in Arabic. Handbook of Arabic literacy: Insights and perspectives. Language and literacy series.

[B37-behavsci-16-00654] Friedmann N., Haluts N., Levy D. (2021). Dysnumeria in sign language: Impaired construction of decimal structures in reading multidigit numbers in a deaf ISL signer. Frontiers in Psychology.

[B38-behavsci-16-00654] Friedmann N., Rahamim E. (2007). Developmental letter position dyslexia. Journal of Neuropsychology.

[B39-behavsci-16-00654] Friedmann N., Rahamim E. (2014). What can reduce letter migrations in letter position dyslexia?. Journal of Research in Reading.

[B40-behavsci-16-00654] Friedmann N., Szterman R. (2006). Syntactic movement in orally-trained children with hearing impairment. Journal of Deaf Studies and Deaf Education.

[B41-behavsci-16-00654] Friedmann N., Szterman R. (2011). The comprehension and production of Wh-questions in deaf and hard-of-hearing children. Journal of Deaf Studies and Deaf Education.

[B42-behavsci-16-00654] Friedmann N., Szterman R., Haddad-Hanna M., Costa J., Castro A., Lobo M., Pratas F. (2010b). The comprehension of relative clauses and Wh questions in Hebrew and Palestinian Arabic hearing impairment. Language acquisition and development: Generative approaches to language acquisition.

[B43-behavsci-16-00654] Funnell E. (1983). Phonological processes in reading: New evidence from acquired dyslexia. British Journal of Psychology.

[B44-behavsci-16-00654] Geraci C. (2015). The status of sign languages.

[B45-behavsci-16-00654] Gori S., Facoetti A. (2015). How the visual aspects can be crucial in reading acquisition: The intriguing case of crowding and developmental dyslexia. Journal of Vision.

[B46-behavsci-16-00654] Güven S., Friedmann N. (2019). Developmental letter position dyslexia in Turkish, a morphologically rich and orthographically transparent language. Frontiers in Psychology—Language Sciences.

[B47-behavsci-16-00654] Güven S., Friedmann N. (2022). Even in predictable orthographies: Surface dyslexia in Turkish. Scientific Studies of Reading.

[B48-behavsci-16-00654] Haddad-Hanna M. (2025). Reading with our Arabic fingerspelling: The first book of reading instruction with sign language.

[B49-behavsci-16-00654] Haddad-Hanna M., Friedmann N. (2009). The comprehension of syntactic structures by Palestinian Arabic-speaking individuals with hearing impairment. Language and Brain.

[B50-behavsci-16-00654] Haluts N., Friedmann N. (2019). SIMBA: A battery for assessment of phonological memory and phonological output buffer in ISL.

[B51-behavsci-16-00654] Haluts N., Friedmann N. (2026). What affects phonological working memory in deaf native signers. Journal of Experimental Psychology: Learning, Memory, and Cognition.

[B52-behavsci-16-00654] Haluts N., Friedmann N. (in press). The manifestation of developmental Phonological Output Buffer impairment in sign language. Rivista di Grammatica Generativa.

[B53-behavsci-16-00654] Haluts N., Levy D., Friedmann N. (2025). Bimodal aphasia and dysgraphia: Phonological output buffer aphasia and orthographic output buffer dysgraphia in spoken and sign language. Cortex.

[B54-behavsci-16-00654] Hannagan T., Amedi A., Cohen L., Dehaene-Lambertz G., Dehaene S. (2015). Origins of the specialization for letters and numbers in ventral occipitotemporal cortex. Trends in Cognitive Sciences.

[B55-behavsci-16-00654] Hanson V. L. (1981). When a word is not the sum of its letters: Fingerspelling and spelling. Haskins Laboratories Status Report on Speech Research.

[B56-behavsci-16-00654] Haptonstall-Nykaza T. S., Schick B. (2007). The transition from fingerspelling to English print: Facilitating English decoding. Journal of Deaf Studies and Deaf Education.

[B57-behavsci-16-00654] Harris M., Terlektsi E., Kyle F. E. (2017). Concurrent and longitudinal predictors of reading for deaf and hearing children in primary school. The Journal of Deaf Studies and Deaf Education.

[B58-behavsci-16-00654] Hirsh-Pasek K. (1986). Beyond the great debate: Fingerspelling as an alternative route to word identification for deaf or dyslexic readers. The Reading Teacher.

[B59-behavsci-16-00654] Holt J. A. (1993). Stanford achievement test-8th edition: Reading comprehension subgroups results. American Annalls of Deaf.

[B60-behavsci-16-00654] Joo S. J., White A. L., Strodtman D. J., Yeatman J. D. (2018). Optimizing text for an individual’s visual system: The contribution of visual crowding to reading difficulties. Cortex.

[B61-behavsci-16-00654] Kohnen S., Nickels L., Castles A., Friedmann N., McArthur G. (2012). When ‘slime’ becomes ‘smile’: Developmental letter position dyslexia in English. Neuropsychologia.

[B62-behavsci-16-00654] Lederberg A. R., Schick B., Spencer P. E. (2013). Language and literacy development of deaf and hard-of-hearing children: Successes and challenges. Developmental Psychology.

[B63-behavsci-16-00654] Lee B., Ortega S. E., Martinez P. M., Midgley K. J., Holcomb P. J., Emmorey K. (2025). Neural associations between fingerspelling, print, and signs: An ERP priming study with deaf readers. Brain and Language.

[B64-behavsci-16-00654] Luckner J. L., Handley C. M. (2008). A summary of the reading comprehension research undertaken with students who are deaf or hard of hearing. American Annals of the Deaf.

[B65-behavsci-16-00654] MacGlaughlin H. M. (2018). The role of fingerspelling in early communication, language, and literacy acquisition of deaf children. Doctoral dissertation.

[B66-behavsci-16-00654] Marschark M., Sapere P., Convertino C. M., Mayer C., Wauters L., Sarchet T. (2009). Are deaf students’ reading challenges really about reading?. American Annals of the Deaf.

[B67-behavsci-16-00654] Marshall J. C., Newcombe F. (1973). Patterns of paralexia: A psycholinguistic approach. Journal of Psycholinguistic Research.

[B68-behavsci-16-00654] Marshall J. C., Newcombe F. (1984). Putative problems and pure progress in neuropsychological single-case studies. Journal of Clinical and Experimental Neuropsychology.

[B69-behavsci-16-00654] Martelli M., Di Filippo G., Spinelli D., Zoccolotti P. (2009). Crowding, reading, and developmental dyslexia. Journal of Vision.

[B70-behavsci-16-00654] McDougall P., Borowsky R., MacKinnon G. E., Hymel S. (2005). Process dissociation of sight vocabulary and phonetic decoding in reading: A new perspective on surface and phonological dyslexias. Brain and Language.

[B71-behavsci-16-00654] Medina J., Fischer-Baum S. (2017). Single-case cognitive neuropsychology in the age of big data. Cognitive Neuropsychology.

[B72-behavsci-16-00654] Meir I., Sandler W. (2007). A language in space: The story of Israeli Sign Language.

[B73-behavsci-16-00654] Miller P., Banado-Aviran E., Hetzroni O. E. (2021). Developing reading skills in prelingually deaf preschool children: Fingerspelling as a strategy to promote orthographic learning. Journal of Deaf Studies and Deaf Education.

[B74-behavsci-16-00654] Navarro G. (2001). A guided tour to approximate string matching. ACM Computing Surveys.

[B75-behavsci-16-00654] Nickels L., Fischer-Baum S., Best W. (2022). Single case studies are a powerful tool for developing, testing and extending theories. Nature Reviews Psychology.

[B76-behavsci-16-00654] Padden C., Gunsauls D. C. (2003). How the alphabet came to be used in a sign language. Sign Language Studies.

[B77-behavsci-16-00654] Padden C., Ramsey C. (1998). Reading ability in signing deaf children. Topics in Language Disorders.

[B78-behavsci-16-00654] Potier Watkins C., Dehaene S., Friedmann N. (2023). Characterizing different types of developmental dyslexias in French: The Malabi Screener. Cognitive Neuropsychology.

[B79-behavsci-16-00654] Puente A., Alvarado J. M., Herrera V. (2006). Fingerspelling and Sign Language as alternative codes for reading and writing words for Chilean deaf signers. American Annals of the Deaf.

[B80-behavsci-16-00654] Quer J., Steinbach M. (2019). Handling sign language data: The impact of modality. Frontiers in Psychology.

[B81-behavsci-16-00654] Rezlescu C., Danaila I., Miron A., Amariei C. (2020). More time for science: Using testable to create and share behavioral experiments faster, recruit better participants, and engage students in hands-on research. Progress in Brain Research.

[B82-behavsci-16-00654] Schirmer B. R., McGough S. M. (2005). Teaching reading to children who are deaf: Do the conclusions of the National Reading Panel apply?. Review of Educational Research.

[B83-behavsci-16-00654] Sehyr Z. S., Petrich J., Emmorey K. (2016). Fingerspelled and printed words are recoded into a speech-based code in short-term memory. The Journal of Deaf Studies and Deaf Education.

[B84-behavsci-16-00654] Shallice T. (1979). Case study approach in neuropsychological research. Journal of Clinical Neuropsychology.

[B85-behavsci-16-00654] Shallice T. (1988). From neuropsychology to mental structure.

[B86-behavsci-16-00654] Shum J., Hermes D., Foster B. L., Dastjerdi M., Rangarajan V., Winawer J., Miller K. J., Parvizi J. (2013). A brain area for visual numerals. Journal of Neuroscience.

[B87-behavsci-16-00654] Spinelli D., De Luca M., Judica A., Zoccolotti P. (2002). Crowding effects on word identification in developmental dyslexia. Cortex.

[B88-behavsci-16-00654] Stein J. (2019). The current status of the magnocellular theory of developmental dyslexia. Neuropsychologia.

[B89-behavsci-16-00654] Sternberg T., Friedmann N. (2007). Developmental graphemic buffer dyslexia. Language and Brain.

[B90-behavsci-16-00654] Sternberg T., Friedmann N. (2009). Are there separate graphemic buffers for reading and writing?. Language and Brain.

[B91-behavsci-16-00654] Stokoe W. C. (2005). Sign language structure: An outline of the visual communication systems of the American deaf. Journal of Deaf Studies and Deaf Education.

[B92-behavsci-16-00654] Stone A., Kartheiser G., Hauser P. C., Petitto L. A., Allen T. E. (2015). Fingerspelling as a novel gateway into reading fluency in deaf bilinguals. PLoS ONE.

[B93-behavsci-16-00654] Sutton-Spence R., Woll B. (1999). The linguistics of British Sign Language: An introduction.

[B94-behavsci-16-00654] Szterman R., Friedmann N. (2020). The effect of syntactic impairment on errors in reading aloud: Text Reading and comprehension of deaf and hard of hearing children. Brain Sciences.

[B95-behavsci-16-00654] Toledano L., Friedmann N. (2023). Letter migrations between words in reading aloud can result either from an impairment in orthographic input or in phonological output. Brain Sciences.

[B96-behavsci-16-00654] Tomazin M. O., Radošević T., Hrastinski I. (2025). Linguistic skills and text reading comprehension in prelingually deaf readers: A systematic review. Journal of Speech, Language, and Hearing Research.

[B97-behavsci-16-00654] Traficante D., Luzzatti C., Friedmann N. (2024). Multiple types of developmental dyslexias in a shallow orthography: Principles for diagnostic screening in Italian. Brain Sciences–Neurolinguistics.

[B98-behavsci-16-00654] Traxler C. (2000). The Stanford Achievement Test, 9th edition: National norming and performance standards for deaf and hard-of-hearing students. The Journal of Deaf Studies and Deaf Education.

[B99-behavsci-16-00654] Tucci S. L., Trussell J. W., Easterbrooks S. R. (2014). A review of the evidence on strategies for teaching children who are DHH grapheme–phoneme correspondence. Communication Disorders Quarterly.

[B100-behavsci-16-00654] Volpato F., Adani F. (2009). The subject/object relative clause asymmetry in Italian hearing-impaired children: Evidence from a comprehension task. Studies in Linguistics.

[B101-behavsci-16-00654] Waters D., Campbell R., Capek C. M., Woll B., David A. S., McGuire P. K., Brammer M. J., MacSweeney M. (2007). Fingerspelling, signed language, text and picture processing in deaf native signers: The role of the mid-fusiform gyrus. Neuroimage.

[B102-behavsci-16-00654] Wauters L. N., Van Bon W. H., Tellings A. E. (2006). Reading comprehension of Dutch deaf children. Reading and Writing.

[B103-behavsci-16-00654] Williams J., Darcy I., Newman S. (2015). Fingerspelling and print processing similarities in deaf and hearing readers. Journal of Language and Literature.

[B104-behavsci-16-00654] Willmes K. (1990). Statistical methods for a single-case study approach to aphasia therapy research. Aphasiology.

[B105-behavsci-16-00654] Willmes K., Gurd J., Kischka U. (2010). The methodological and statistical foundations of neuropsychological assessment. The handbook of clinical neuropsychology.

